# High-performance web services for querying gene and variant annotation

**DOI:** 10.1186/s13059-016-0953-9

**Published:** 2016-05-06

**Authors:** Jiwen Xin, Adam Mark, Cyrus Afrasiabi, Ginger Tsueng, Moritz Juchler, Nikhil Gopal, Gregory S. Stupp, Timothy E. Putman, Benjamin J. Ainscough, Obi L. Griffith, Ali Torkamani, Patricia L. Whetzel, Christopher J. Mungall, Sean D. Mooney, Andrew I. Su, Chunlei Wu

**Affiliations:** Department of Molecular and Experimental Medicine, The Scripps Research Institute, 10550 North Torrey Pines Road, La Jolla, CA 92037 USA; Current address: Avera Cancer Institute, 11099 North Torrey Pines Road, La Jolla, CA 92037 USA; Department of Biomedical Informatics and Medical Education, The University of Washington, Box SLU-BIME 358047, Seattle, WA 98195 USA; McDonnell Genome Institute, Washington University School of Medicine, 4444 Forest Park Ave, St. Louis, MO 63108 USA; Department of Integrative Structural and Computational Biology, The Scripps Research Institute, 10550 North Torrey Pines Road, La Jolla, CA 92037 USA; The Scripps Translational Science Institute, The Scripps Research Institute, 10550 North Torrey Pines Road, La Jolla, CA 92037 USA; Center for Research in Biological Systems, University of California San Diego, 9500 Gilman Drive, La Jolla, CA 92093 USA; Lawrence Berkeley National Laboratory, 1 Cyclotron Road, Berkeley, CA 94720 USA

**Keywords:** Annotation, Gene, Variant, API, Cloud, Repository, Database

## Abstract

Efficient tools for data management and integration are essential for many aspects of high-throughput biology. In particular, annotations of genes and human genetic variants are commonly used but highly fragmented across many resources. Here, we describe MyGene.info and MyVariant.info, high-performance web services for querying gene and variant annotation information. These web services are currently accessed more than three million times permonth. They also demonstrate a generalizable cloud-based model for organizing and querying biological annotation information. MyGene.info and MyVariant.info are provided as high-performance web services, accessible at http://mygene.info and http://myvariant.info. Both are offered free of charge to the research community.

**Electronic supplementary material:**

The online version of this article (doi:10.1186/s13059-016-0953-9) contains supplementary material, which is available to authorized users.

## Background

The accumulation of biomedical knowledge is growing exponentially. There has been tremendous effort to structure research findings as annotations on biological entities (e.g., genes, genetic variants, and pathways). However, these annotations are fragmented among many resources that range greatly in terms of size, funding, and visibility (see, e.g., Ensembl [1], UniProt [2], PROSITE [3], and Reactome [4]). Tools for knowledge integration enable more efficient analysis of genome-scale data sets and discovery of relationships between biological entities.

Bioinformaticians facing data integration problems generally pursue one of two strategies: data warehousing or data federation. Data warehousing involves downloading flat files from various sources, writing parsers to process the files, and then loading the parsed data into a local database. This strategy has the advantage of very high performance, but it also requires significant initial effort to write the parsers and ongoing effort to keep the resource up to date. On the other hand, data federation works by accessing remote data resources through web services. Federated data solutions are always up to date, but extra care is required to maintain the links, and large queries may take a long time to return due to server and network limitations. Moreover, the dependability of federated solutions is entirely dependent on the stability of the remote resources.

## Results and Discussion

Here we present an alternative solution for integrating annotations on genes and human variants. MyGene.info and MyVariant.info are open source, high-performance, and continuously updated data application programming interfaces (APIs) for accessing comprehensive, structured gene and variant annotations. These resources are offered as cloud-based web service endpoints with the goal of providing “annotation as a service.” MyGene.info and MyVariant.info are centralized repositories for aggregating and serving dispersed annotation data. Both are free of charge for use by the research community.

Other centralized resources for gene and variant annotations currently exist for genes (e.g., Bioconductor AnnotationData Packages [5] and Biomart [6]) and variants (e.g., ANNOVAR [7]). Relative to these existing tools, MyGene.info and MyVariant.info have several advantages. First, a local database is not required, reducing setup, administration, and maintenance costs. Second, we provide a high-performance API that allows real-time queries in analysis pipelines or web applications.

### Data integration

**Fig. 1:**
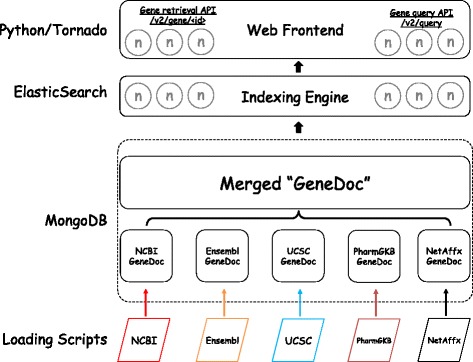
Schematic design of the MyGene.info architecture. Colors depict different update frequencies. Small gray circles indicate multiple nodes for scalability. MyVariant.info shares the same architecture as MyGene.info except for different sets of annotation data sources and update frequencies. Additional file 2: Figure S1 shows the exact architecture of MyVariant.info

MyGene.info is maintained as a comprehensive and up-to-date repository for gene annotations. It integrates data from large, centralized databases as well as smaller, more specialized sources. Each data source has its own data importer, converting external data sources to a list of objects in JavaScript Object Notation (JSON) format. Each individual JSON object uses the National Center for Biotechnology (NCBI) gene ID [8] as the preferred primary key. The output of each parser is stored in a MongoDB instance with a timestamp recorded for each individual annotation object, and then all objects with the same primary key are combined into a single annotation object. In addition, we have built a scheduling system that automates the updates for each data source according to its own schedule (see Fig. 1). Currently, MyGene.info provides more than 200 gene-specific annotation fields ([9] and Additional file 1: Table S1) covering more than 13 million genes for more than 15,000 species [10].

MyVariant.info is built with a similar design and architecture as MyGene.info, but it focuses specifically on annotations of human genetic variants (Additional file 2: Figure S1). We utilize the nomenclature from the Human Genome Variation Society (HGVS) [11] to define the primary key in MyVariant.info (see Methods for specific rules). To prevent incorrect usage of HGVS IDs that could lead to potential errors in clinical interpretation, we also developed and implemented a variant validation function to ensure that all variant IDs included in MyVariant.info strictly follow HGVS guidelines. Currently, MyVariant.info contains more than 500 variant-specific annotation types ([12] and Additional file 3: Table S2) from dozens of resources, covering more than 334 million unique variants [13], including both coding and non-coding variants.

### Web services

Performance, scalability, and stability are three key features of a successful web service provider. We built an Elasticsearch-based cluster to index the underlying JSON objects for both MyGene.info and MyVariant.info. This indexing engine provides both superior query performance and rich query syntax to handle a large amount of concurrent queries for a variety of use cases. The Elasticsearch cluster also comes with inherent scalability, so that we can dynamically adjust the size of the cluster to accommodate increased bandwidth as needed. For example, the MyGene.info system is currently hosted on the Amazon EC2 platform with four moderate servers, which based on our tests, can handle traffic from >5000 concurrent users for approximately 10,000 requests per minute. Greater than 95 % of actual user requests take less than 30 ms to process (Additional file 2: Figure S2). This dynamic cluster setup also promotes stability of these services by allowing us to perform maintenance on individual nodes without bringing down the whole system.

Since the release of the v2 API (July 2013), MyGene.info has accumulated more than 133 million requests, and it currently averages more than 3 million requests per month. Primary users include public resources like BioGPS [14], the Monarch Initiative [15], and CIViC [16]. In addition, numerous individual users incorporate MyGene.info into their bioinformatics analysis pipelines. According to our usage monitoring, approximately 30 % of traffic comes from our BioGPS application, while 70 % of traffic comes externally from more than 6000 unique IP addresses. The MyVariant.info API was launched in June 2015. To date, MyVariant.info has accumulated more than 1.5 million user queries.

### Use case

To demonstrate their utility, we used MyVariant.info and MyGene.info to reimplement a typical analysis pipeline for interpreting exome sequencing results and identifying candidate genes for a rare Mendelian disease. In 2010, Ng et al. identified *DHODH* mutations as the genetic cause for Miller syndrome [17]. In their exome analysis, genes with nonsynonymous (NS) variants, splice acceptor and donor site mutations (SS), and coding indels (I) were first identified. Next, they filtered for genes containing NS/SS/I variants in all four sequenced samples. Previously observed variants in dbSNP129 [18], the 1000 Genomes Project [19], or HapMap were excluded. PolyPhen predictions [20] were used to prioritize variants that were predicted to be damaging. This process undoubtedly involved downloading, parsing, and analyzing annotation data from multiple databases, representing a significant investment of time and effort.

**Fig. 2:**
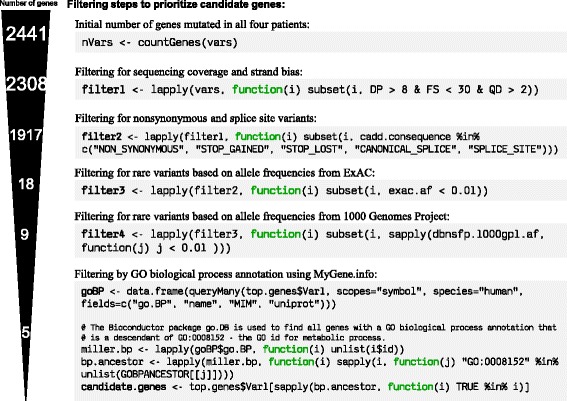
The demo workflow for candidate gene prioritization using MyVariant.info and MyGene.info web services. We reimplemented five filtering steps in this workflow to prioritize candidate genes from a Miller syndrome study [17]. Selected R code is displayed for each filter step, using *myvariant* and *mygene* Bioconductor packages. The number of candidate genes left at each filtering step is displayed at the left side. The full code is available at https://github.com/sulab/myvariant.info/blob/master/docs/ipynb/myvariant_R_miller.ipynb, and also in Additional file 2: Supplementary Note 1

Using the MyGene.info and MyVariant.info R packages alone, we are able to implement an updated version of this pipeline: about 50 lines of code, requiring no local installation of variant annotation databases or software tools (see Fig. 2, [21], and Additional file 2: Supplementary Note 1). We first filtered for NS/SS/I variants and removed variants observed in the 1000 Genomes Project (as in [17]). We also incorporated an allele frequency filter based on data from the Exome Aggregation Consortium [22], filtered for candidate genes involved in metabolic processes (“GO:0008152”) based on Gene Ontology annotations, and ranked candidate genes based on Combined Annotation Dependent Depletion (CADD) score [23] (an estimate of pathogenicity). After implementing this workflow for the Miller syndrome study, we were left with only five candidate genes, including the causal gene *DHODH*. In addition, since MyVariant.info contains comprehensive and up-to-date variant annotations, it offers users the flexibility to further tailor this workflow based on other annotation fields (e.g., SIFT score [24], PolyPhen score [20], and clinical significance from ClinVar [25], etc.).

The utility of MyGene.info and MyVariant.info extends beyond this particular pipeline for exome sequencing analysis. Users can search for genes or variants using a wide variety of identifiers, and annotations can be retrieved for either single entities or lists. Users can also perform data-dependent queries (e.g., to find all variants with ExAC allele frequencies below 0.05 in the gene *BRCA1*). Queries can be performed through the web-based API or by using data access libraries for R or Python. These tools are flexible enough to incorporate into custom workflows, as well as responsive enough to perform real-time queries from other web applications. Finally, although MyGene.info and MyVariant.info focus on genes and human genetic variants, the underlying open source infrastructure is easily extensible to any type of biological entity.

## Implementation

### Data sources

Currently, MyGene.info contains data for about 200 annotation fields ([9], Additional file 1: Table S1) that were retrieved from eight public databases (Table 1), and MyVariant.info contains data for about 500 annotation fields ([12], Additional file 3: Table S2) that were retrieved from 14 public databases (Table 2). Our scheduler checks every data resource on a weekly basis, detects any changes to the source files, and applies incremental updates to our live servers. Annotation data from different databases exist in different formats, e.g., VCF, XML, and TSV. We wrote an individual data parser for all annotation data sources. Data parsers automatically import data from raw sources to facilitate regular updates where possible. All parser code for MyGene.info is available at Bitbucket [26]. All parser code for MyVariant.info is available at GitHub [27]. We also included sequence-based annotations generated from SnpEff [28] for each variant (if available). For example, multiple transcripts overlapping with a variant will be included as a list under the “snpeff.ann” field.

**Table 1:** The list of data sources for MyGene.info. Column 1 lists the names of all eight data sources included in MyGene.info. Column 2 lists the version of each data source. Column 3 lists the URL for each data source

Source	Version	URL	Reference
NCBI Entrez	2015-10-24	http://www.ncbi.nlm.nih.gov/gquery/	[40]
Ensembl	82	http://www.ensembl.org/	[1]
UniProt	2015-10-15	http://www.uniprot.org/	[2]
NetAffx	na35	https://www.affymetrix.com/analysis/index.affx	[41]
PharmGKB	2015-10-05	https://www.pharmgkb.org/	[42]
UCSC	2015-10-20	https://genome.ucsc.edu/	[43]
CPDB	31	http://cpdb.molgen.mpg.de/CPDB	[44]
RefSeq	68	http://www.ncbi.nlm.nih.gov/refseq/	[45]

**Table 2:** The list of data sources for MyVariant.info. Column 1 lists the names of all 14 data sources included in MyVariant.info. Column 2 lists the version of each data source. Column 3 shows the number of variants from each data source included in MyVariant.info. Column 4 lists the URL for each data source

Source	Version	No. of variants	URL	Reference
dbNSFP	v3.0c	82,030,830	https://sites.google.com/site/jpopgen/dbNSFP	[46]
dbSNP	v144	145,132,257	http://www.ncbi.nlm.nih.gov/snp/	[18]
ClinVar	2015-09	114,627	http://www.ncbi.nlm.nih.gov/clinvar/	[25]
EVS	v2	1,977,300	http://evs.gs.washington.edu/EVS/	[47]
CADD	v1.2	163,690,986	http://cadd.gs.washington.edu/	[23]
MutDB	-	420,221	http://www.mutdb.org/	[48]
GWAS Catalog	From UCSC	15,243	http://www.ebi.ac.uk/gwas/	[49]
COSMIC	v68 from UCSC	1,024,498	http://cancer.sanger.ac.uk/cosmic/	[50]
DOCM	-	1119	http://docm.genome.wustl.edu/	[51]
SNPedia	-	5907	http://www.snpedia.com/index.php/SNPedia	[52]
EMVClass	-	12,066	http://geneticslab.emory.edu/emvclass/emvclass.php	[53]
Wellderly	-	21,240,519	http://www.stsiweb.org/wellderly/	[54]
ExAC	v0.3	10,195,872	http://exac.broadinstitute.org/	[22]
GRASP	v2.0.0.0	2,212,148	http://grasp.nhlbi.nih.gov/Overview.aspx	[55]

### Data integration

The output of each data parser is a list of JSON objects. Each object contains an ‘_id’ field as the primary key, which uniquely identifies a biological entity. MyGene.info uses the NCBI gene ID [8] as the preferred primary key, although the Ensembl gene ID [1] is used when no mapping to the NCBI gene ID is available.

For primary keys for variants, we used the nomenclature defined by the Human Genome Variation Society (HGVS) [11], as it is a recommended and widely accepted standard for describing variants. HGVS nomenclature allows multiple names to describe the same variant based on different reference sequences (e.g., genome assembly, transcript, or protein sequences). To define unique primary keys, we use the HGVS names based on the most commonly used reference genome assembly (currently hg19) and use *chr1*, *chr2*, *…*, *chr22*, *chrX*, *chrY*, and *chrMT* to represent chromosomes (e.g., chr11:g.111959693G>T — more examples are given in Additional file 2: Table S3). Although the primary keys of variant objects are based on genomic reference sequences, other valid HGVS names corresponding to alternate reference sequences (e.g., NC_000011.9:g.111959693G>T, NM_003002.2:c.274G>T) are also stored in each variant object and are indexed for queries.

We implemented a scheduling system to automate the updates for each data source. Currently, both MyGene.info and MyVariant.info are updated weekly. The output of each parser is stored in a MongoDB instance with a timestamp recorded for each individual annotation object, and all objects with the same primary key (‘_id’ field) are combined into a single annotation object (see the examples in Additional file 2: Figure S3). This setup is advantageous, as it ensures the independent processing of each annotation source. Any single failure in the update process will not break the entire merging process, as in the case when a source file format changes and breaks a data parser. In that case, the last successful version of that failed source will be used until the parser is adapted to the changes.

After the merging process, each JSON object contains all variant annotations aggregated from multiple sources. We then use Elasticsearch to index all fields within an annotation object so that users can make queries to retrieve annotations for their relevant genes or variants. Elasticsearch is a highly scalable, open source, full-text search and analytics engine. It provides a rich query syntax and inherent scalability to handle large-scale data queries in real time.

### Application programming interfaces

On top of Elasticsearch, we built REST-based web services using the Tornado web framework. Tornado is a Python-based web framework built upon asynchronous networking technology that can provide tens of thousands of concurrent connections with a moderate server.

MyGene.info provides two simple-to-use REST-based web services: a gene query service and a gene annotation service. The gene query service allows users to query for gene annotations using any identifier or keyword, while the gene annotation service provides a convenient way to retrieve gene-centric annotations when gene IDs (NCBI gene IDs or Ensembl gene IDs) are available.

MyVariant.info also provides two REST-based web services: a variant query service that returns matching variant objects based on user queries and a variant retrieval service that returns the matching variant object(s) for a given ID (HGVS names, RS IDs, etc.).

Batch mode is supported by both services for querying a large list of IDs or query terms in one request. Both query services provide rich query syntax suitable for a variety of use cases. For example, users can query for matching variant annotation objects by various criteria, such as genomic ranges, prediction score cutoffs, exact field matching, and keyword search in a text field. Users also have the option of specifying the subset of fields they want to return if they do not require the entire annotation object. More complicated queries can be constructed by combining multiple query terms with Boolean operators or by conducting faceting, with aggregations for special use cases. Information on the types of queries that are enabled by MyGene.info can be found at[29]. Information on the types of queries that are enabled by MyVariant.info can be found at [30].

### Use case demonstration

We reanalyzed the exome sequencing data generated by Ng et al. for their Miller syndrome study [17]. Genomic DNA from four patients were sequenced in this study. FASTQ files were processed according to the GATK best practice [29]. Individual samples were aligned to the hg19 reference genome using BWA 0.7.10. Variants were called using GATK 3.3 HaplotypeCaller, and quality scores were recalibrated using GATK VariantRecalibrator. The Bioconductor packages *myvariant* and *mygene* were utilized to demonstrate a streamlined application for variant filtering and prioritization of candidate genes in rare Mendelian disorders.

### Query interface

Although the initial design of MyGene.info and MyVariant.info APIs is targeted to bioinformatics developers, we also consider it a necessity to provide a query interface for our APIs, which can serve both as the demo interface for developers and the entry points for general researchers. We have implemented the initial versions of our easy-to-use query interface for both APIs: http://mygene.info/demo and http://myvariant.info/demo. They can be easily accessed from each site’s landing page.

### Python and R clients

A Python client and an R client are available for both MyGene.info and MyVariant.info. The Python client for MyGene.info can be downloaded at the Python Package Index [30].

The R client for MyGene.info is released as part of Bioconductor [31]. The Python client for MyVariant.info can be downloaded at the Python Package Index [32], and the R client for MyVariant.info is also released as part of Bioconductor [33].

### Availability and requirements

MyGene.info web services can be accessed at http://mygene.info, with the interactive API documentation at http://mygene.info/v2/api/and the full documentation at http://docs.mygene.info.

MyVariant.info web services can be accessed at http://myvariant.info, with the interactive API documentation at http://myvariant.info/v1/api/and the full documentation at http://docs.myvariant.info.

MyGene.info and MyVariant.info are both open source projects (licensed under the Apache License, Version 2.0). The source code for these projects can be found at [34] (MyGene.info web frontend), [35] (MyGene.info data backend), and [36] (MyVariant.info). They have also been deposited to Zenodo (https://zenodo.org/) with assigned DOIs: 10.5281/zenodo.48146 (MyGene.info web frontend) [37], 10.5281/zenodo.48145 (MyGene.info data backend) [38], and 10.5281/zenodo.48086 (MyVariant.info) [39].

Exome sequence data from two siblings with Miller syndrome and two unrelated affected individuals were provided by Ng et al. [17] through the database of Genotypes and Phenotypes (dbGaP) under accession number [dbGaP:phs000244.v1.p1].

## Conclusions

MyGene.info (http://mygene.info) and MyVariant.info (http://myvariant.info) are provided as highperformance web services for querying gene and variant annotation information, currently with over three million user requests per month.

## Additional files

Additional file 1: Table S1. The gene-specific annotation fields available from MyGene.info. (XLS 32 kb) Additional file 2: Includes Figure S1: Schematic design of MyVariant.info; Figure S2: Histograms of the request time for MyGene.info; Figure S3: JSON annotation object examples; Table S3: Examples of HGVS nomenclature; Supplementary Note 1: IPython notebook for Miller syndrome study. (PDF 946 kb) Additional file 3: Table S2. The variant-specific annotation fields available from MyVariant.info. (XLS 58 kb)
