# Supplementary material for: High-performance web services for querying gene and variant annotation
Source: Genome Biol. 2016 May 6;17:91. doi: 10.1186/s13059-016-0953-9 (PMC4858870; doi:10.1186/s13059-016-0953-9)
Supplement: Additional file 2: — Includes Figure S1: Schematic design of MyVariant.info; Figure S2: Histograms of the request time for MyGene.info; Figure S3: JSON annotation object examples; Table S3: Examples of HGVS nomenclature; Supplementary Note 1: IPython notebook for Miller syndrome study. (PDF 946 kb) [file 13059_2016_953_MOESM2_ESM.pdf]

Additional file 2

Figure S1. Schematic design of the MyVariant.info architecture. Colors depict different update frequencies. Small grey circles indicate multiple nodes for scalability.

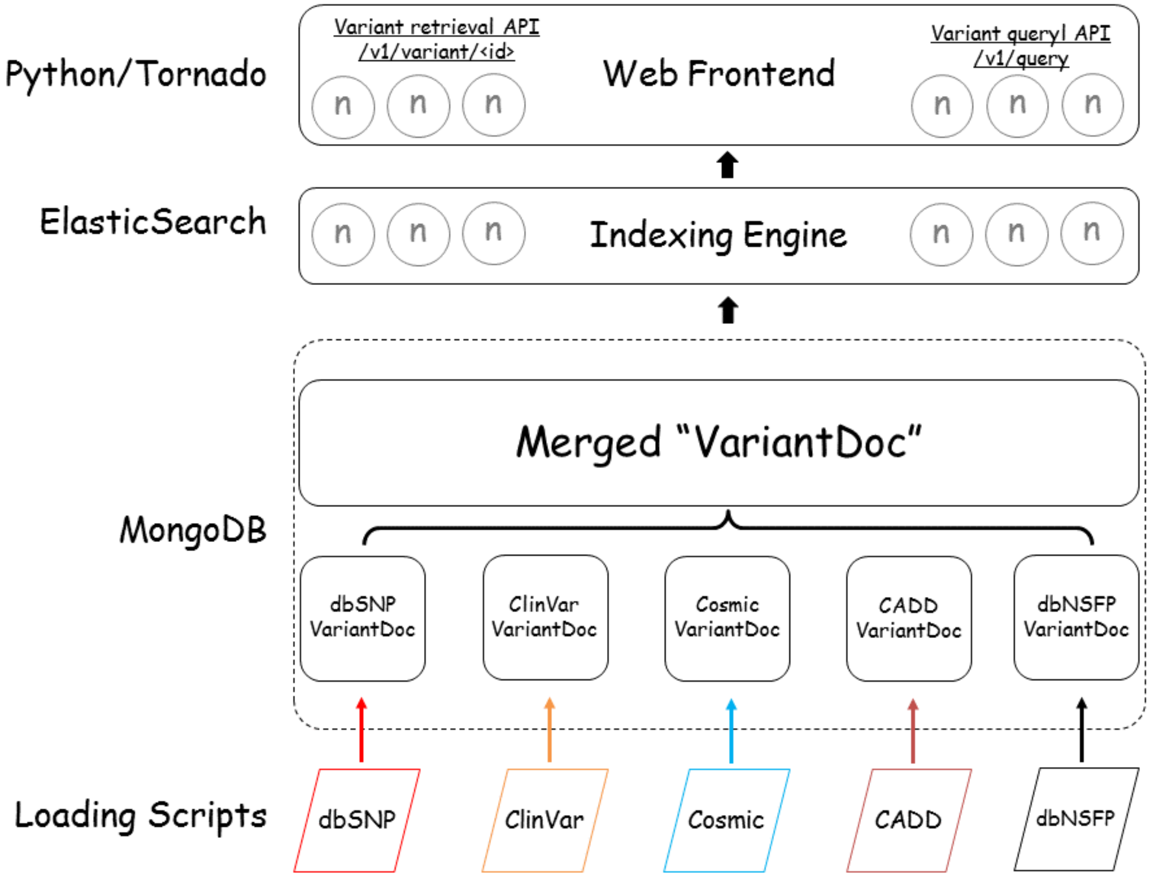

Figure S2. Histograms of the request time in millisecond for actual user requests to the gene query service (top) and the gene annotation service (bottom) of MyGene.info. The number on each bar indicates the actual count. The data were extracted from the logs of server nodes during recent 30-day period (08/01/2015-09/01/2015).

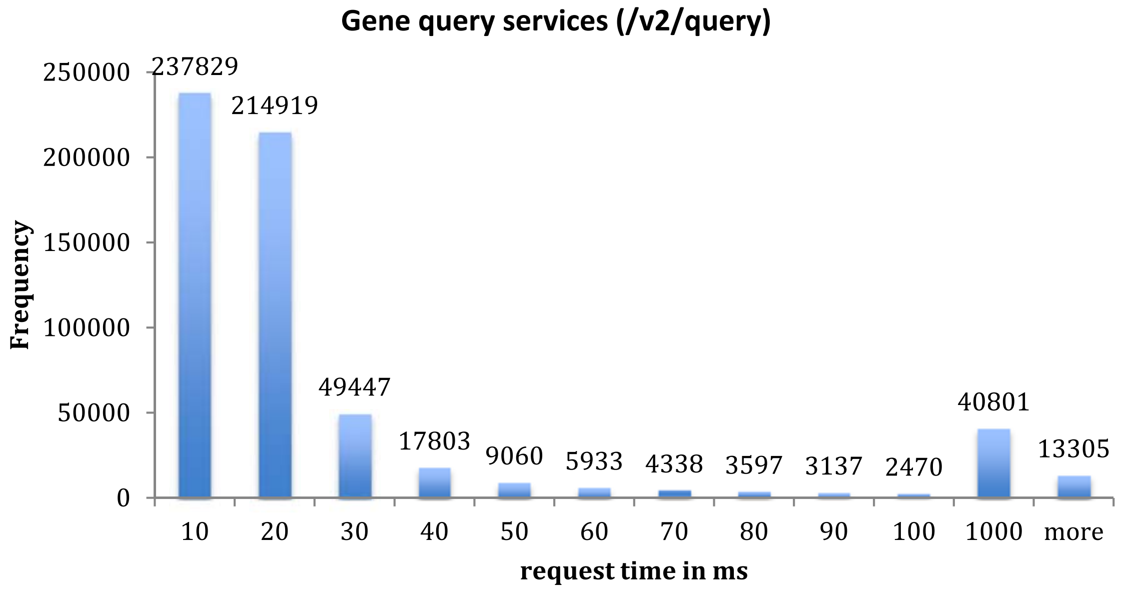

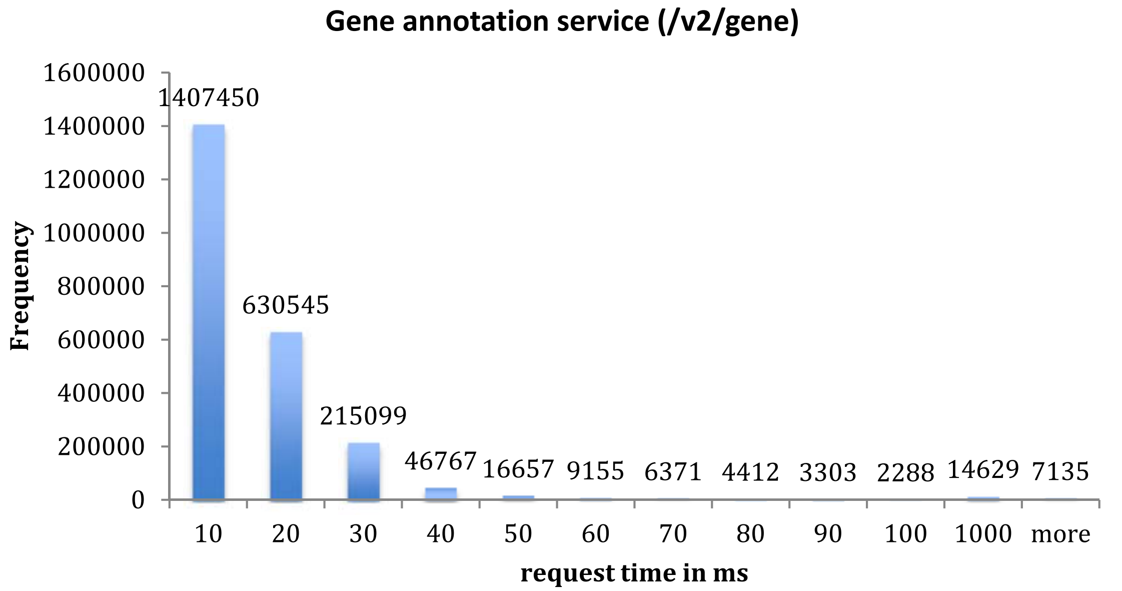

Figure S3. Two examples of JSON annotation objects stored and indexed at a) MyGene.info and b) MyVariant.info. Some contents are collapsed and the full objects can be viewed at the links above. Additional gene and variant query examples can be found at: https://gist.github.com/kevinxin90/7edb38903ff997b5d670 (MyGene.info) and https://gist.github.com/kevinxin90/b8e88cbcf23d45479ede (MyVariant.info).

a) http://MyGene.info/v2/gene/**7157**


```
{
   _id: "7157",
 ▸ accession: {…},
 ▸ alias: […],
 ▸ ensembl: {…},
   entrezgene: 7157,
 ▸ exons: {…},
 ▸ exons_hg19: {…},
 ▸ generif: […],
 ▸ genomic_pos: {…},
 ▴ genomic_pos_hg19: {…},
 ▸ go: {…},
   HGNC: "11998",
 ▸ homologene: {…},
   HPRD: "01859",
 ▸ interpro: […],
 ▸ ipi: […],
   map_location: "17p13.1",
   MIM: "191170",
   name: "tumor protein p53",
 ▸ pathway: {…},
 ▸ pdb: […],
 ▸ pfam: […],
   pharmgkb: "PA36679",
   pir: "A25224",
 ▸ reagent: {…},
 ▸ refseq: {…},
 ▸ reporter: {…},
 ▸ retired: […],
   summary: "This gene encodes a tumor
   suppressor protein containing
   transcriptional activation, DNA binding,
   ...",
   symbol: "TP53",
   taxid: 9606,
   type_of_gene: "protein-coding",
 ▸ unigene: […],
 ▸ uniprot: {…},
   Vega: "OTTHUMG00000162125",
 ▸ wikipedia: {…}
}
```


b) http://MyVariant.info/v1/variant/**chr7:g.55241707G>T**


```
{
   _id: "chr7:g.55241707G>T",
   _version: 1,
 ▸ cadd: {…},
 ▸ clinvar: {…},
 ▾ cosmic: {
      alt: "T",
      chrom: "7",
      cosmic_id: "COSM6253",
    ▾ hg19: {
         end: 55241707,
         start: 55241707
      },
      mut_freq: 0.04,
      mut_nt: "G>T",
      ref: "G",
      tumor_site: "lung"
   },
 ▸ dbnsfp: {…},
 ▸ dbsnp: {…},
 ▸ docm: {…},
 ▸ mutdb: {…},
 ▸ snpedia: {…},
 ▸ snpeff: {…},
 ▸ vcf: {…}
}
```

Table S1. Gene-specific annotation fields available from MyGene.info web services. The first column is the field name; the second column indicates if the field is indexed (therefore it can be queried on); the third column is the data type of the field (like string, integer, object, etc.).

(See provided supplementary excel file: **Supplementary table 1_mygene_fields.xlsx**)

Table S2. Variant-specific annotation fields available from MyVariant.info web services. The first column is the field name; the second column indicates if the field is indexed (therefore it can be queried on); the third column is the data type of the field (like string, integer, object, etc.).

(See provided supplementary excel file: **Supplementary table 2_myvariant_fields.xlsx**)

Table S3. Examples of HGVS (Human Genome Variation Society) nomenclature.

**Variant types**	**HGVS nomenclature**	**Notes**
Substitution	chr1:g.241T>C	single nucleotide substitution
Deletion	chr1:g.413del	single nucleotide deletion
	chr1:g.290_297del	>1 nucleotide deletion
Duplication	chr1:g.413dup	single nucleotide duplication
	chr1:g.692_694dup	several nucleotide duplication
Insertion	chr1:g.451_452insT	single nucleotide insertion
	chr1:g.451_452insGAGA	several nucleotide insertion
	chr1:g.777_778insAB012345.1	large insertion with a submitted sequence
Inversion	chr1:g.1077_1080inv	short inversion
	chr1:g.1458_oXYZ:457inv	large inversion

Supplementary note 1. This is the print out of the jupyter notebook at: https://github.com/sulab/myvariant.info/blob/master/docs/ipynb/myvariant_R_miller.ipynb

MyVariant.info and MyGene.info Use Case

The following R script demonstrates the utility of the **MyVariant.info** and **MyGene.info** R clients to annotate variants and prioritize candidate genes in patients with rare Mendelian diseases. This specific study uses data obtained from the database of phenotype and genotype (dbGaP) study. FASTQ files generated by Ng et al for the Miller syndrome study (http://www.ncbi.nlm.nih.gov/pubmed/19915526) were processed according to the Broad Institute's best practices. Individual samples were aligned to the hg19 reference genome using BWA-MEM 0.7.10. Variants were called using GATK 3.3-0 HaplotypeCaller and quality scores were recalibrated using GATK VariantRecalibrator.

Initial Library Imports and Data Loading

In [1]:

```
library(myvariant, quietly=TRUE)
library(mygene, quietly=TRUE)
library(VariantAnnotation, quietly=TRUE)
library(GO.db, quietly=TRUE)
source("https://raw.githubusercontent.com/SuLab/myvariant.info/master/docs/ipynb/mendelian.R")
setwd("~/sulab/myvariant/vcf/recal")
vcf.files <- paste(getwd(), list.files(getwd()), sep="/")
```

```
Attaching package: ‘BiocGenerics’

The following objects are masked from ‘package:parallel’:

    clusterApply, clusterApplyLB, clusterCall, clusterEvalQ,
    clusterExport, clusterMap, parApply, parCapply, parLapply,
    parLapplyLB, parRapply, parSapply, parSapplyLB

The following object is masked from ‘package:stats’:

    xtabs

The following objects are masked from ‘package:base’:

    anyDuplicated, append, as.data.frame, as.vector, cbind, colnames,
    do.call, duplicated, eval, evalq, Filter, Find, get, intersect,
    is.unsorted, lapply, Map, mapply, match, mget, order, paste, pmax,
    pmax.int, pmin, pmin.int, Position, rank, rbind, Reduce, rep.int,
    rownames, sapply, setdiff, sort, table, tapply, union, unique,
    unlist, unsplit

Creating a generic function for ‘nchar’ from package ‘base’ in package ‘S4Vectors’

Attaching package: ‘VariantAnnotation’

The following object is masked from ‘package:base’:

    tabulate

Welcome to Bioconductor

    Vignettes contain introductory material; view with
    'browseVignettes()'. To cite Bioconductor, see
    'citation("Biobase")', and for packages 'citation("pkgname")'.

Loading required package: DBI


Attaching package: ‘plyr’

The following object is masked from ‘package:XVector’:

    compact

The following object is masked from ‘package:IRanges’:

    desc

The following object is masked from ‘package:S4Vectors’:

    rename
```

`vcf.files` contains paths to the vcf files for each of the four patients included in this analysis. Exome sequence data from two sibs with Miller syndrome and two unrelated affected individuals used in this vignette was provided by Ng et al. (2010) Nature Genetics (phs000244.v1.p1) (http://www.ncbi.nlm.nih.gov/pubmed/19915526). As this is protected information, access must be requested from dbGaP here (http://www.ncbi.nlm.nih.gov/projects/gap/cgi-bin/study.cgi?study_id=phs000244.v1.p1) in order to run this notebook.

mendelian.R defines some helper functions that are used in the analysis occurring after annotation retrieval:

`replaceWith0` - replaces all NAs in a data.frame with 0.

`rankByCaddScore` - for prioritizing genes by deleteriousness (scaled CADD score).

Annotating variants with MyVariant.info

The following function reads in each output VCF file using the VariantAnnotation package available from Bioconductor. Install with `biocLite("VariantAnnotation")`. `formatHgvs` (from the ***myvariant*** Bioconductor package) is a function that reads the genomic location and variant information from the VCF to create HGVS IDs which serve as a primary key for each variant. The function `getVariants` makes the queries to MyVariant.info to retrieve annotations.

In [2]:
```
getVars <- function(vcf.file){
  cat(paste("Processing ", vcf.file, "...\n", sep=" "))
  vcf <- readVcf(vcf.file, genome="hg19")
  vcf <- vcf[isSNV(vcf)]
  vars <- rowRanges(vcf)
  vars <- as(vars, "DataFrame")
  vars$query <- formatHgvs(vcf, "snp")
  annotations <- getVariants(vars$query, fields=c("dbnsfp.genename", "dbnsfp.1000gp1.af",
                                                  "exac.af", "cadd.consequence", "cadd.phred"), verbose=FALSE)
  annotations[c('DP', 'FS', 'QD')] <- info(vcf)[c('DP', 'FS', 'QD')]
  annotations <- replaceWith0(annotations)
  annotations <- subset(annotations, !(dbnsfp.genename %in% c("NULL", 0)))
  annotations
}

vars <- lapply(vcf.files, getVars)
```

```
Processing  /Users/cyrusafrasiabi/recal/subject01_recalibrate_SNP_vqsr.vcf ...
found header lines for 3 ‘fixed’ fields: ALT, QUAL, FILTER
found header lines for 24 ‘info’ fields: AC, AF, ..., VQSLOD, culprit
found header lines for 5 ‘geno’ fields: GT, AD, DP, GQ, PL

Concatenating data, please be patient.

Processing  /Users/cyrusafrasiabi/recal/subject02_recalibrate_SNP_vqsr.vcf ...
found header lines for 3 ‘fixed’ fields: ALT, QUAL, FILTER
found header lines for 24 ‘info’ fields: AC, AF, ..., VQSLOD, culprit
found header lines for 5 ‘geno’ fields: GT, AD, DP, GQ, PL

Concatenating data, please be patient.

Processing  /Users/cyrusafrasiabi/recal/subject03_recalibrate_SNP_vqsr.vcf ...
found header lines for 3 ‘fixed’ fields: ALT, QUAL, FILTER
found header lines for 24 ‘info’ fields: AC, AF, ..., VQSLOD, culprit
found header lines for 5 ‘geno’ fields: GT, AD, DP, GQ, PL

Concatenating data, please be patient.

Processing  /Users/cyrusafrasiabi/recal/subject04_recalibrate_SNP_vqsr.vcf ...
found header lines for 3 ‘fixed’ fields: ALT, QUAL, FILTER
found header lines for 24 ‘info’ fields: AC, AF, ..., VQSLOD, culprit
found header lines for 5 ‘geno’ fields: GT, AD, DP, GQ, PL

Concatenating data, please be patient.
```

All genes (variants with a valid `dbnsfp.genename`) that are mutated amongst all four patients are examined. The following function counts the number of genes in `inp` that are mutated among all four patients:

In [3]:
```
countGenes <- function(inp) {
    ret <- subset(data.frame(table(unlist(lapply(inp, function(i) unique(i$dbnsfp.genename))))),
    Freq == 4)
    cat("Genes remaining: ", paste(nrow(ret)))
    ret
}
```

Initial Number of Genes Mutated in All Patients

In [4]:
```
nVars <- countGenes(vars)
```

```
Genes remaining:  2441
```

In [5]:
```
filter1 <- lapply(vars, function(i) subset(i, DP > 8 & FS < 30 & QD > 2))

nFilter1 <- countGenes(filter1)
```

```
Genes remaining:  2308
```

2 - Filtering for Nonsynonymous and Splice Site Variants

Mendelian diseases are most likely to be caused by nonsynonymous mutations. The CADD database annotates the mutation type in the field "cadd.consequence".

In [6]:
```
filter2 <- lapply(filter1, function(i) subset(i, cadd.consequence %in% c("NON_SYNONYMOUS", "STOP_GAINED", "STOP_
                                          "CANONICAL_SPLICE", "SPLICE_SITE")))

nFilter2 <- countGenes(filter2)
```

```
Genes remaining:  1917
```

3 - Filtering for Allele Frequency Annotated by ExAC

The third filter keeps rare variants according to the ExAC data set with allele frequency < 0.01. Rare diseases are likely caused by mutations that have not been documented yet.

In [7]:
```
filter3 <- lapply(filter2, function(i) subset(i, exac.af < 0.01))

nFilter3 <- countGenes(filter3)
```

```
Genes remaining:  18
```

4 - Filtering for Allele Frequency Annotated by 1000 Genomes Project

The fourth filter keeps rare variants according to the 1000 Genomes Project with allele frequency < 0.01.

In [8]:
```
filter4 <- lapply(filter3, function(i) subset(i, sapply(dbnsfp.1000gp1.af, function(j) j < 0.01 )))

top.genes <- countGenes(filter4)
```

```
Genes remaining:  9
```

5 - Filtering by GO Biological Process Annotation using MyGene.info

Since Miller Syndrome is known to be an inborn error of metabolism, this filter keeps only genes involved in metabolic processes according to their GO biological process annotation. To accomplish this, GO biological process annotations are pulled for each remaining gene using the **MyGene.info** R client, which can be installed from Bioconductor (`biocLite("mygene")`). Here, the `queryMany` function is used, requesting the necessary annotations using the `fields` parameter.

In [9]:
```
goBP <- data.frame(queryMany(top.genes$Var1, scopes="symbol", species="human", fields=c("go.BP", "name", "MIM",
```

```
Finished
```

The Bioconductor package **go.DB** is used to find all genes with a GO biological process annotation that is a descendant of `GO:0008152` - the GO id for metabolic process.

In [10]:
```
miller.bp <- lapply(goBP$go.BP, function(i) unlist(i$id))
bp.ancestor <- lapply(miller.bp, function(i) sapply(i, function(j) "GO:0008152" %in% unlist(GOBPANCESTOR[[j]])))
candidate.genes <- top.genes$Var1[sapply(bp.ancestor, function(i) TRUE %in% i)]
cat("Genes remaining: ", length(candidate.genes))
```

```
Genes remaining:  5
```

Prioritizing genes

The remaining five genes can be prioritized according to CADD (deleteriousness) score. `rankByCaddScore` extracts the average CADD scores of the variants in each gene and ranks in descending order.

In [11]:
```
ranked <- rankByCaddScore(candidate.genes, filter4)
ranked
```

Out[11]:

	gene	cadd.phred
**1**	DHODH	26.81
**2**	CTBP2	21.385
**3**	PIK3R3	20.7
**4**	CDC27	18.545
**5**	CDON	10.02

This analysis highlights the use of the **MyVariant.info** and **MyGene.info** annotation services to narrow the candidate gene list from 2441 genes to 5 - representing a significant decrease in the burden of manual biological analysis.
